# Endoplasmic reticulum chaperone BiP/GRP78 knockdown leads to autophagy and cell death of arginine vasopressin neurons in mice

**DOI:** 10.1038/s41598-020-76839-z

**Published:** 2020-11-12

**Authors:** Yohei Kawaguchi, Daisuke Hagiwara, Takashi Miyata, Yuichi Hodai, Junki Kurimoto, Hiroshi Takagi, Hidetaka Suga, Tomoko Kobayashi, Mariko Sugiyama, Takeshi Onoue, Yoshihiro Ito, Shintaro Iwama, Ryoichi Banno, Valery Grinevich, Hiroshi Arima

**Affiliations:** 1 Department of Endocrinology and Diabetes, Nagoya University Graduate School of Medicine, 65 Tsurumai-cho, Showa-ku, Nagoya, 466-8550 Japan; 2 Department of Neuropeptide Research in Psychiatry, Central Institute of Mental Health, Medical Faculty Mannheim, University of Heidelberg, 68159 Mannheim, Germany; 3 Research Center of Health, Physical Fitness and Sports, Nagoya University, Nagoya, 464-8601 Japan

**Keywords:** Neuroscience, Endocrinology

## Abstract

The immunoglobulin heavy chain binding protein (BiP), also referred to as 78-kDa glucose-regulated protein (GRP78), is a pivotal endoplasmic reticulum (ER) chaperone which modulates the unfolded protein response under ER stress. Our previous studies showed that BiP is expressed in arginine vasopressin (AVP) neurons under non-stress conditions and that BiP expression is upregulated in proportion to the increased AVP expression under dehydration. To clarify the role of BiP in AVP neurons, we used a viral approach in combination with shRNA interference for BiP knockdown in mouse AVP neurons. Injection of a recombinant adeno-associated virus equipped with a mouse AVP promoter and BiP shRNA cassette provided specific BiP knockdown in AVP neurons of the supraoptic (SON) and paraventricular nuclei (PVN) in mice. AVP neuron-specific BiP knockdown led to ER stress and AVP neuronal loss in the SON and PVN, resulting in increased urine volume due to lack of AVP secretion. Immunoelectron microscopy of AVP neurons revealed that autophagy was activated through the process of AVP neuronal loss, whereas no obvious features characteristic of apoptosis were observed. Pharmacological inhibition of autophagy by chloroquine exacerbated the AVP neuronal loss due to BiP knockdown, indicating a protective role of autophagy in AVP neurons under ER stress. In summary, our results demonstrate that BiP is essential for the AVP neuron system.

## Introduction

The endoplasmic reticulum (ER) plays an essential role in synthesis, folding, assembly and transport of secretory and transmembrane proteins^1,2^ which account for one third of the total proteins in humans^3^. Disturbance of ER homeostasis causes the accumulation of misfolded proteins in the ER lumen leading to ER stress^4^. The unfolded protein response (UPR) is a cellular defense mechanism by which ER folding capacity is upregulated^5^ and protein load is decreased in the ER^6^. The UPR is thus primarily a protective mechanism; however, it will cause cell death if ER stress is severe and prolonged, and will also eliminate damaged cells which could be potentially harmful to surrounding cells and tissues^7^.

The immunoglobulin heavy chain binding protein (BiP), also referred to as the 78-kDa glucose-regulated protein (GRP78), is one of the most abundant ER chaperones^8–10^. BiP binds to newly synthesized polypeptides to promote their folding and also binds to misfolded proteins to facilitate correct refolding and prevent their aggregation^11^. In addition, BiP is a pivotal modulator of UPR signaling^12^, which under non-stress conditions suppresses UPR activity by binding to three transmembrane ER stress transducers: protein kinase RNA-like ER kinase, inositol-requiring protein 1, and activating transcription factor 6 (ATF6). Since BiP preferentially binds to misfolded and unfolded proteins, it dissociates from the ER stress transducers under conditions of ER stress, leading to UPR activation^13,14^. It was also shown that BiP expression is increased by ER stress^11,15,16^, and has been used as a general indicator of ER stress in addition to the UPR^17^.

Arginine vasopressin (AVP), an antidiuretic hormone that promotes water reabsorption in the kidney, is mainly synthesized in the magnocellular neurons of the supraoptic (SON) and paraventricular nuclei (PVN) in the hypothalamus^18^. AVP mRNA expression in the SON and PVN is relatively high, and AVP synthesis and release are upregulated by only a 1–2% increase in plasma osmolality^19^, suggesting that AVP neurons must meet a large demand for AVP production as specialized secretory cells. AVP precursors are subjected to proper folding in the ER^20,21^, and through the folding process, some degree of AVP precursors undergo ER-associated degradation (ERAD)^22^. Furthermore, knockout of the Sel1L-Hrd1 protein complex, a principal ER-resident E3 ligase in mammalian ERAD, is reported to cause marked retention and aggregation of AVP precursors in the ER, resulting in polyuria due to AVP deficiency^23^. These data indicate that ER protein quality control is essential for appropriate AVP synthesis and release. Indeed, ER stress has been implicated in the pathophysiology of some genetic types of central diabetes insipidus such as familial neurohypophysial diabetes insipidus (FNDI) which is caused by the accumulation of mutant AVP precursors in the ER^24–30^.

Our previous studies showed that BiP is expressed in AVP neurons under non-stress conditions and that its expression is upregulated in proportion to the increase in AVP expression under dehydration^31^. Furthermore, we also demonstrated that AVP release is impaired in dehydrated ATF6α knockout mice in which BiP is not properly upregulated in response to ER stress^29^. While these data suggest that BiP might be involved in the synthesis and release of AVP, the role of BiP in the AVP neuron system has not been fully clarified.

In the present study, we specifically ablated BiP expression in AVP neurons by utilizing virally-mediated shRNA interference and analyzed the morphology of AVP neurons as well as fluid homeostasis in mice.

## Results

### Validation of recombinant adeno-associated virus (rAAV) vectors and BiP shRNA in mouse AVP neurons

**Figure 1:**
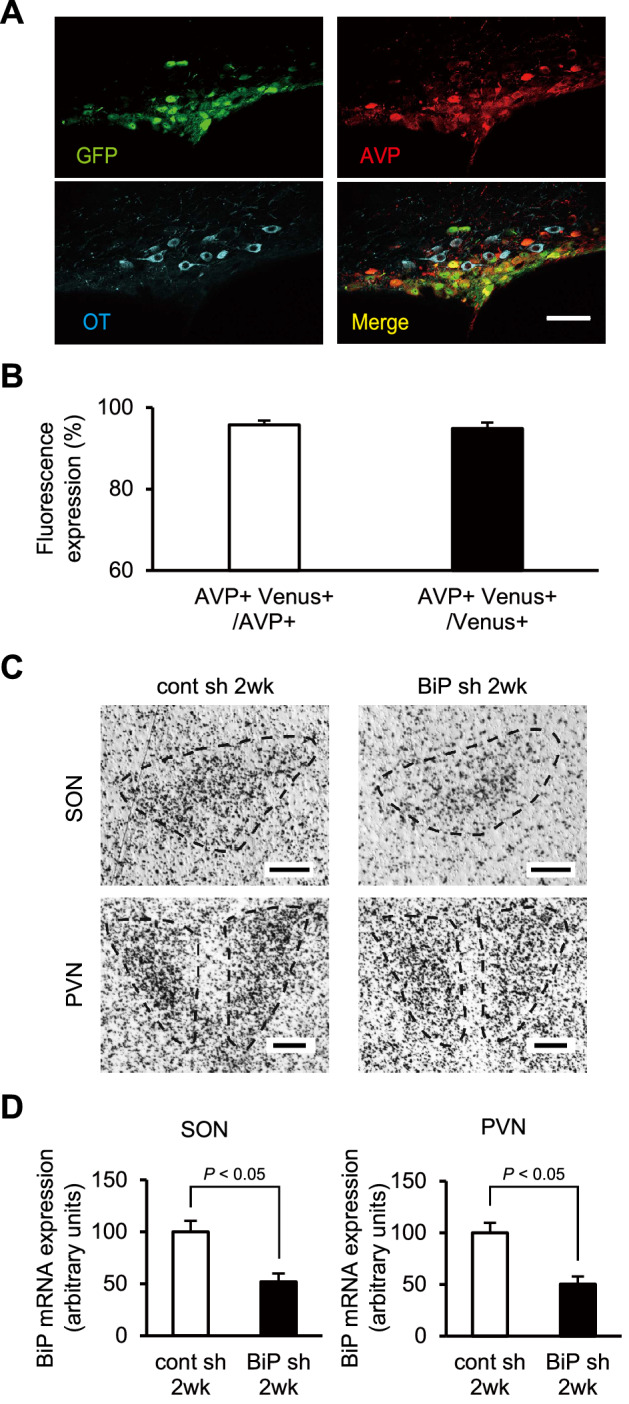
Validation of rAAV vectors and BiP shRNA in mouse AVP neurons. **(A)** Representative images of immunofluorescence staining to enhance the Venus signal (green), and to detect AVP (magenta) and OT (cyan) in the SON 2 weeks after rAAV-AVPp-Venus injection. Scale bar: 50 μm. **(B)** The proportion of both AVP and Venus-immunoreactive (ir) cells to AVP-ir (AVP + Venus + /AVP +) and Venus-ir cells (AVP + Venus + /Venus +) in the SON 2 weeks after rAAV-AVPp-Venus injection. **(C)** Representative images of in situ hybridization for BiP mRNA in the SON and PVN 2 weeks after injection of rAAV-AVPp-scrambled shRNA (cont sh 2wk) and rAAV-AVPp-BiP shRNA (BiP sh 2wk). Scale bars: 100 μm. **(D)** Expression levels of BiP mRNA in the SON and PVN in the cont sh 2wk and BiP sh 2wk groups. Mean expression levels of BiP mRNA in the SON and PVN in the cont sh 2wk group are expressed as 100. Results were analyzed by an unpaired Student’s *t*-test and are expressed as the means ± SE (**B**, *n* = 6; **D**, *n* = 7 per group).

For the validation of our rAAV vectors in mouse AVP neurons, we injected rAAV carrying an AVP promoter and Venus cDNA (rAAV-AVPp-Venus) into the SON and analyzed the proportion of Venus-expressing cells by immunostaining for Venus, AVP and oxytocin (OT) 2 weeks after virus injection (Fig. 1A). Results showed that 95.7% of AVP neurons (314/328 cells) expressed Venus, and 94.3% of Venus-positive cells (314/333 cells) were AVP neurons (Fig. 1B). In contrast, only 1.5% of Venus-positive cells (5/333 cells) expressed OT, which included Venus-positive cells simultaneously expressing AVP and OT (4/333 cells). These data demonstrate that our rAAVs carrying the AVP promoter were almost completely restricted to AVP neurons in mice, as shown in rats^32^.

To specifically ablate BiP in AVP neurons, rAAVs harboring the AVP promoter sequence followed by BiP shRNA were employed. We injected rAAV-AVPp-BiP shRNA into the bilateral SON and PVN for BiP knockdown in AVP neurons and rAAV-AVPp-scrambled shRNA as a control. The knockdown efficiency 2 weeks after rAAV-AVPp-BiP shRNA injection was 51.9% in the SON and 50.2% in the PVN, respectively (Fig. 1C,D).

### AVP neuron-specific BiP knockdown increases urine volume

**Figure 2:**
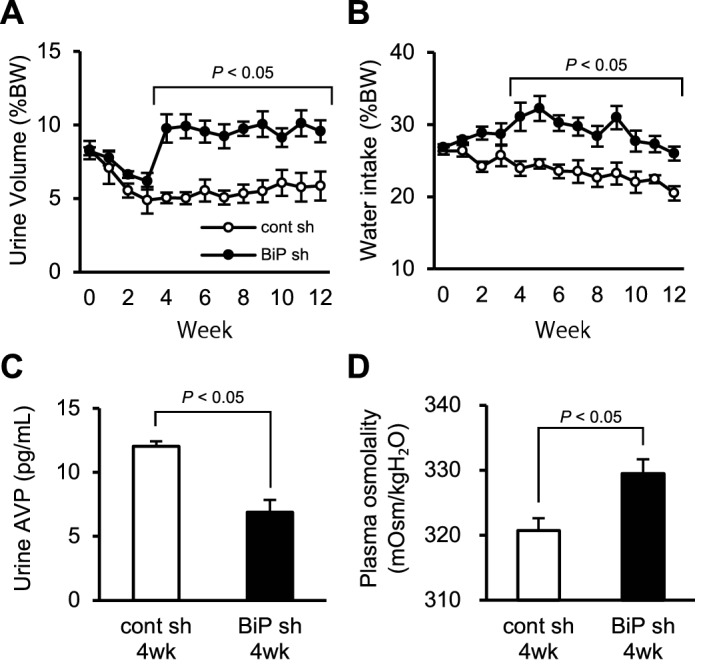
Effect of AVP neuron-specific BiP knockdown on urine volume, water intake, plasma osmolality and urine AVP concentration in mice. Urine volume **(A)** and water intake **(B)** in mice injected with rAAV-AVPp-scrambled shRNA (cont sh, open circles) and rAAV-AVPp-BiP shRNA (BiP sh, closed circles). Urine AVP concentrations **(C)** and plasma osmolality **(D)** at 4 weeks in the cont sh and BiP sh groups. Results were analyzed by two-way ANOVA with repeated measures followed by a Bonferroni test (**A**,**B**) or unpaired Student’s *t*-test (**C**,**D**) and are expressed as the means ± SE (*n* = 4–8 per group).

Next, we compared urine volumes, water intake, body weight, plasma osmolality and urine AVP concentrations between mice injected with rAAV-AVPp-scrambled shRNA and rAAV-AVPp-BiP shRNA into the bilateral SON and PVN up to 12 weeks after virus injection. 4 weeks after virus injection, urine volume (Fig. 2A) as well as water intake (Fig. 2B) increased significantly in mice injected with rAAV-AVPp-BiP shRNA compared with control mice, while no significant differences were observed in body weight (data not shown). Furthermore, urine AVP concentrations were significantly decreased (Fig. 2C) and plasma osmolality was significantly elevated (Fig. 2D) 4 weeks after injection with rAAV-AVPp-BiP shRNA compared to control mice.

### AVP neuron-specific BiP knockdown leads to loss of AVP neurons

**Figure 3:**
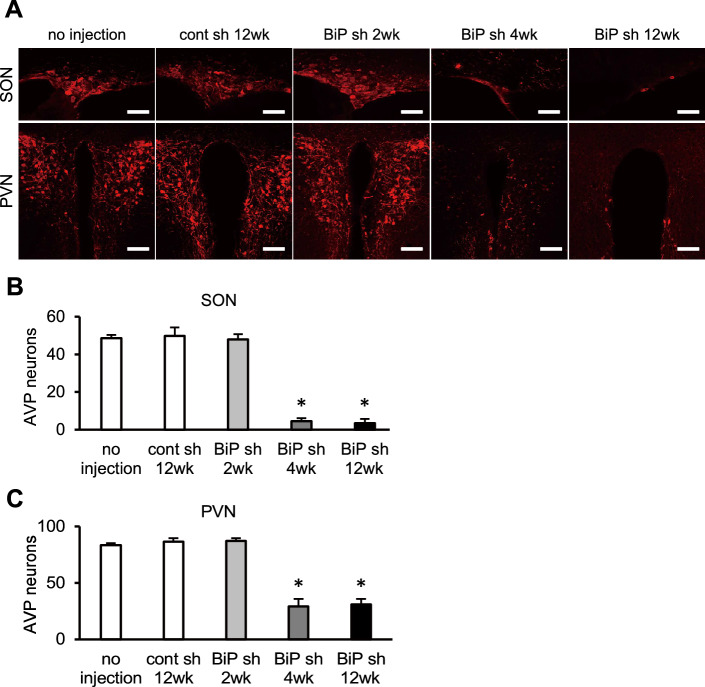
AVP neuronal loss in AVP neuron-specific BiP knockdown mice. (**A**) Representative images of immunofluorescence staining for AVP in the SON and PVN of un-injected mice (no injection), or 12 weeks after injection of rAAV-AVPp-scrambled shRNA (cont sh 12wk), and at 2 (BiP sh 2wk), 4 (BiP sh 4wk) and 12 weeks after rAAV-AVPp-BiP shRNA injection (BiP sh 12wk). Scale bars: 50 μm (SON), 100 μm (PVN). The number of AVP neurons in the SON **(B)** and PVN **(C)** in the un-injected, cont sh 12wk, BiP sh 2wk, BiP sh 4wk and BiP sh 12wk groups. Results were analyzed by one-way ANOVA followed by a Bonferroni test and are expressed as the means ± SE (*n* = 3–6 per group). *P < 0.05 compared with the un-injected group.

To examine the effects of BiP knockdown on AVP neuronal viability, we counted AVP neurons in the SON and PVN of mice injected with rAAV-AVPp-BiP shRNA or rAAV-AVPp-scrambled shRNA into the bilateral SON and PVN as well as un-injected mice (Fig. 3A). There were no significant differences in the number of AVP neurons between the un-injected and scrambled shRNA groups (Fig. 3B,C). In contrast, while mice injected with rAAV-AVPp-BiP shRNA showed no significant changes 2 weeks after injection, AVP neurons were markedly lost 4 and 12 weeks after virus injection; the degree of neuronal loss was similar between these two time points (Fig. 3B,C). These data indicate that AVP neuronal loss occurred between 2 and 4 weeks after rAAV injection and that BiP is essential for the AVP neuron system.

### AVP neuron-specific BiP knockdown leads to ER stress in AVP neurons

**Figure 4:**
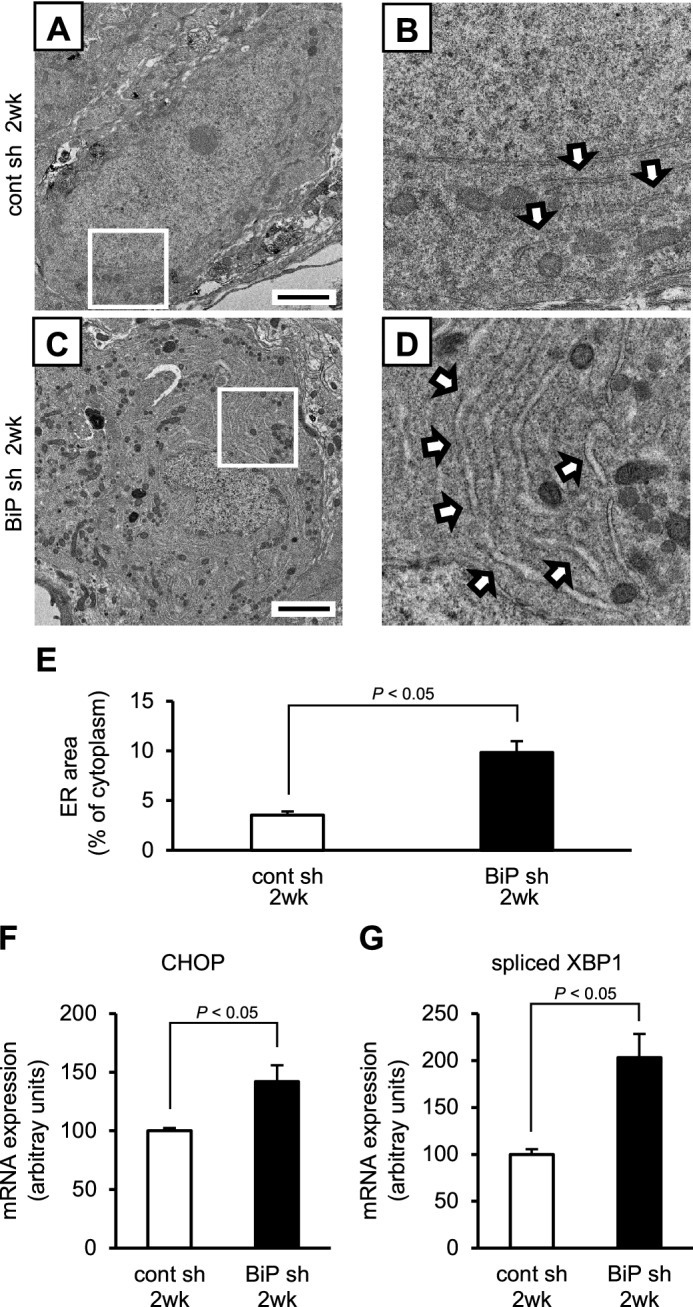
Evaluation of ER stress in AVP neurons of AVP neuron-specific BiP knockdown mice. Representative electron microscopic images of AVP neurons in the SON of mice 2 weeks after injection of rAAV-AVPp-scrambled shRNA (cont sh 2wk, **A**,**B**) and rAAV-AVPp-BiP shRNA (BiP sh 2wk, **C**,**D**). Higher magnification images of the boxed area in (**A**,**C**) are shown in (**B**,**D**). Arrows indicate the ER lumen (**B**,**D**). Scale bars: 2 μm. **(E)** Quantification of ER area relative to the cytoplasm in AVP neurons in the cont sh 2wk and BiP sh 2wk groups. Quantitative real-time RT-PCR analysis for CHOP and spliced XBP1 mRNA in the PVN in the cont sh 2wk **(F)** and BiP sh 2wk groups **(G)**. Mean mRNA expression levels in the cont sh 2wk group are expressed as 100. Results were analyzed by an unpaired Student’s *t*-test and are expressed as the means ± SE (**E**, *n* = 5; **F**, **G**, *n* = 7 per group).

Immunoelectron microscopy revealed that BiP knockdown in AVP neurons led to dilatation of the ER lumen 2 weeks post-injection at a time point when AVP neurons were not yet lost, whereas a normal ER lumen was observed in AVP neurons of control mice injected with rAAV-AVPp-scrambled shRNA (Fig. 4A-D). Quantitative analyses found that the ratio of ER area to cytoplasm was increased in AVP neurons of mice injected with rAAV-AVPp-BiP shRNA compared to control mice (Fig. 4E). The expression of mRNA for the ER stress markers C/EBP homologous protein (CHOP) and spliced X-box binding protein 1 (XBP1) was upregulated in the PVN of BiP shRNA-injected mice 2 weeks after injection (Fig. 4F,G). These data demonstrate that AVP neuron-specific BiP knockdown leads to ER stress in AVP neurons prior to cell death.

### AVP neuron-specific BiP knockdown activates autophagy in AVP neurons through the process of AVP neuronal loss

ER stress and apoptosis are known to be closely related^33^. To investigate the involvement of apoptosis in AVP neuronal loss after BiP knockdown, we performed a terminal deoxynucleotidyl transferase-mediated dUTP nick end-labeling (TUNEL) assay. In the SON and PVN of AVP neuron-specific BiP knockdown mice, there were almost no TUNEL-positive cells either 2 or 4 weeks after BiP shRNA injection (Supplementary Fig. 1).

**Figure 5:**
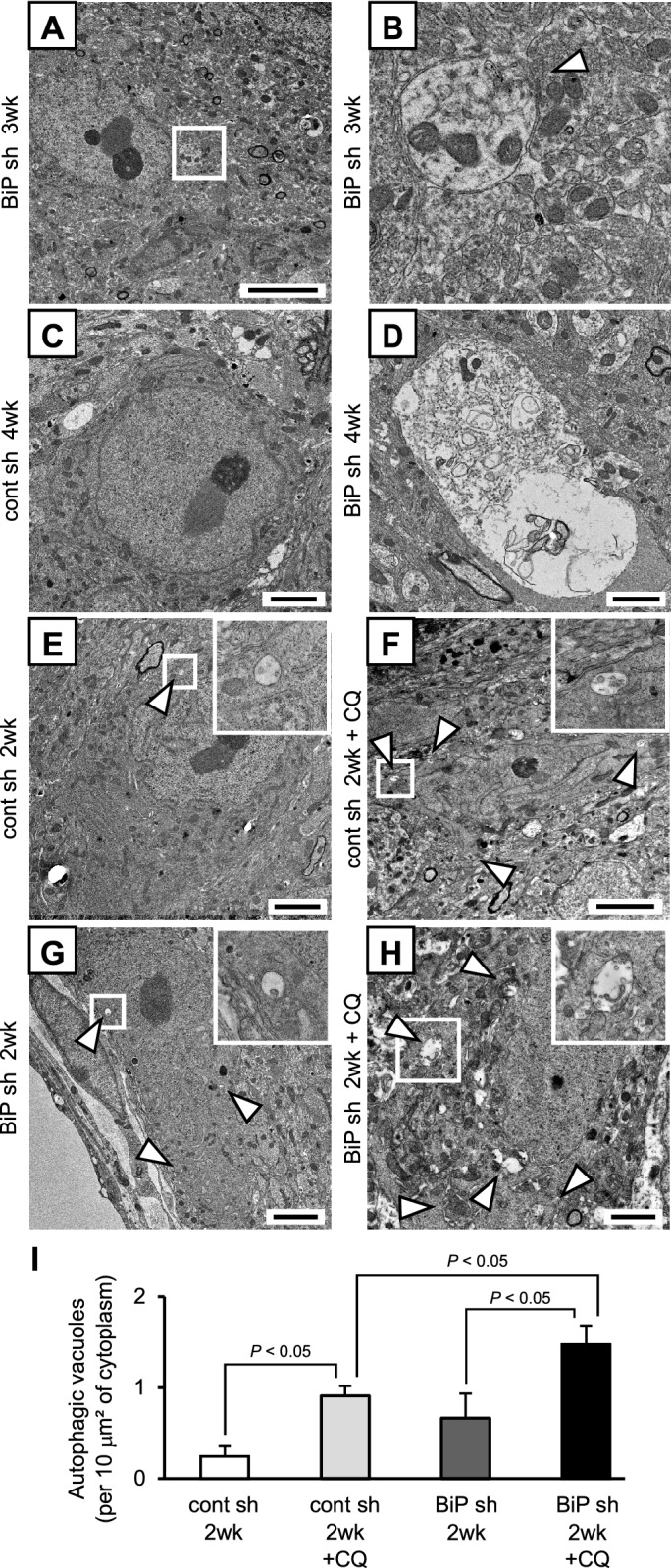
Evaluation of autophagic activity in AVP neurons of AVP neuron-specific BiP knockdown mice. Representative electron microscopic images of AVP neurons in the SON of mice 3 weeks after rAAV-AVPp-BiP shRNA injection (BiP sh 3wk, **A**,**B**) and 4 weeks after injection of rAAV-AVPp-scrambled shRNA (cont sh 4wk, **C**) and rAAV-AVPp-BiP shRNA (BiP sh 4wk, **D**). Higher magnification images of the boxed area in (**A**) are shown in (**B**). Representative electron microscopic images of AVP neurons in the SON of mice 2 weeks after injection of rAAV-AVPp-scrambled shRNA alone (cont sh 2wk, **E**) and with chloroquine treatment (cont sh 2wk + CQ, **F**), and rAAV-AVPp-BiP shRNA alone (BiP sh 2wk, **G**) and with chloroquine treatment (BiP sh 2wk + CQ, **H**). Higher magnification images of the boxed areas are shown in the insets at upper right. **(I)** The number of autophagic vacuoles in AVP neurons in the cont sh 2wk, cont sh 2wk + CQ, BiP sh 2wk and BiP sh 2wk + CQ groups. Arrowheads (**B, E–H**) indicate autophagic vacuoles. Results were analyzed by two-way ANOVA followed by a Bonferroni test and are expressed as the means ± SE (*n* = 7–8 per group). Scale bars: 2 μm.

Given that AVP neuronal loss occurred between 2 and 4 weeks after injection with rAAV-AVPp-BiP shRNA, we performed electron microscopy of AVP neurons 3 and 4 weeks after injection to detect morphological changes in the dying AVP neurons. While nuclear structure was relatively well preserved, autophagic vacuoles containing degraded organelles were observed 3 weeks after BiP shRNA injection (Fig. 5A,B). Furthermore, whereas no obvious morphological changes were found in AVP neurons of control mice 4 weeks after injection with rAAV-AVPp-scrambled shRNA (Fig. 5C), large vacuoles containing various organelles undergoing degradation were detected in some cells by 4 weeks after injection with BiP shRNA (Fig. 5D). For quantification of autophagic activity in AVP neurons under BiP knockdown, we compared the number of autophagic vacuoles in AVP neurons of mice 2 weeks after injection of scrambled or BiP shRNA with or without chloroquine treatment, a lysosomal inhibitor which hampers the autophagic degradation process (Fig, 5E–H). In scrambled shRNA-injected mice, autophagic vacuoles in AVP neurons of chloroquine-treated mice were significantly increased compared to mice without chloroquine administration (Fig. 5I), confirming a constant autophagic flux in AVP neurons and the validity of chloroquine treatment. Autophagic vacuoles in BiP shRNA-injected mice treated with chloroquine were further increased compared to scrambled shRNA-injected mice (Fig. 5I). These results demonstrate that autophagic flux in AVP neurons is activated by AVP neuron specific-BiP knockdown.

### Autophagic inhibition exacerbates AVP neuronal loss in AVP neuron-specific BiP knockdown mice

**Figure 6:**
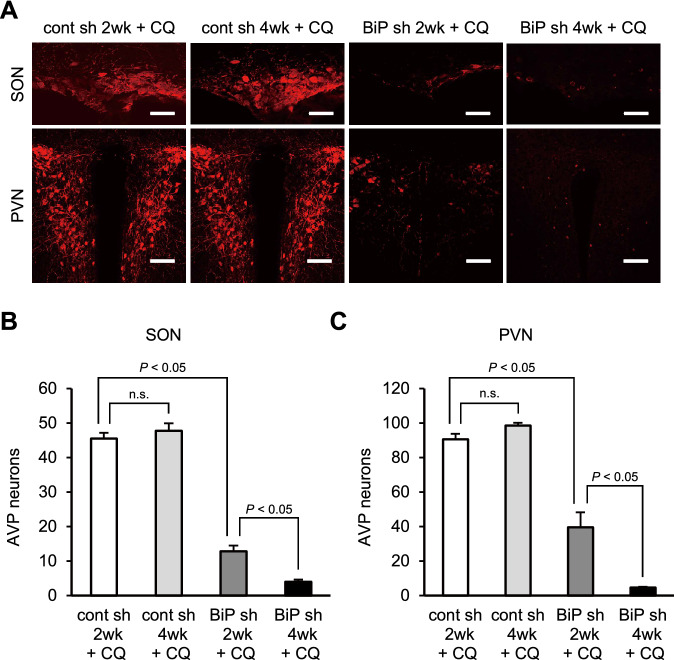
Pharmacological inhibition of autophagy exacerbates AVP neuronal loss in AVP neuron-specific BiP knockdown mice. **(A)** Representative images of immunofluorescence staining for AVP in the SON and PVN of chloroquine-treated mice 2 (cont sh 2wk + CQ) and 4 weeks after injection of rAAV-AVPp-scrambled shRNA (cont sh 4wk + CQ), and 2 (BiP sh 2wk + CQ) and 4 weeks after rAAV-AVPp-BiP shRNA injection (BiP sh 4wk + CQ). Scale bars: 50 μm (SON), 100 μm (PVN). The number of AVP neurons in the SON **(B)** and PVN **(C)** in the cont sh 2wk + CQ, cont sh 4wk + CQ, BiP sh 2wk + CQ and BiP sh 4wk + CQ groups. Results were analyzed by two-way ANOVA followed by a Bonferroni test and are expressed as the means ± SE (*n* = 3 per group). *n.s.* not significant.

To examine the role of autophagy through the process of AVP neuronal loss under AVP neuron-specific BiP knockdown, we counted AVP neurons in the SON and PVN of chloroquine-treated mice injected with rAAV-AVPp-BiP shRNA or rAAV-AVPp-scrambled shRNA into the bilateral SON and PVN (Fig. 6A). There were no significant differences in the number of AVP neurons between chloroquine-treated mice 2 and 4 weeks after scrambled shRNA injection (Fig. 6B,C), and the values were similar to those in un-injected mice (Fig. 3B, C). In contrast, AVP neurons were markedly lost in chloroquine-treated mice 2 weeks after BiP shRNA injection (Fig. 6B,C), at the time point when AVP neurons have not been lost yet in mice injected with BiP shRNA alone (Fig. 3B,C). Moreover, AVP neurons were further decreased in chloroquine-treated mice 4 weeks after BiP shRNA injection (Fig. 6B,C).

### AVP neuron-specific BiP knockdown activates microglia and astrocytes in the SON and PVN

Immunostaining for ionized calcium-binding adaptor molecule 1 (IBA1) and glial fibrillary acidic protein (GFAP) revealed the upregulation of IBA1 and GFAP expression in the SON and PVN of mice 4 weeks after injection with BiP shRNA (Supplementary Fig. 2). The mRNA expression of tumor necrosis factor-α (TNF-α)*,* interleukin (IL)-6 and IL-1β was upregulated in the PVN of BiP shRNA-injected mice after 2 weeks (Supplementary Fig. 3). These data indicate that microglia and astrocytes are activated in response to the death of AVP neurons.

## Discussion

In the present study, we performed BiP knockdown in mouse AVP neurons by injecting rAAV vectors expressing BiP shRNA under the control of an AVP promoter into the SON and PVN. Our data demonstrated that AVP neuron-specific BiP knockdown leads to ER stress and activates autophagy in AVP neurons followed by AVP neuronal loss.

Since BiP whole-body knockout mice were reported to be embryonically lethal due to apoptosis of the inner cell mass of the embryo^34^, BiP conditional knockout has been utilized to investigate the role of BiP in a wide range of specific cell types. Previous studies demonstrated that BiP knockout leads to ER stress and the death of various cell types including hepatocytes^35^, adipocytes^36^, myocytes^37^, respiratory epithelial cells^38,39^, hematopoietic cells^40^, Purkinje cells^41^, oligodendrocytes and Schwann cells^42^. In the current study, we also showed that BiP conditional knockdown in AVP neurons led to dilatation of the ER lumen and upregulation of ER stress markers followed by AVP neuronal loss. Furthermore, it is of note that approximately 90% of AVP neurons in the SON and 70% in the PVN were lost by partial knockdown (about 50%) of BiP expression. This finding contrasts with previous studies in which cell death resulted from complete deletion of BiP expression using a conditional knockout strategy. We previously reported that the basal expression of BiP is relatively high in AVP neurons^31^, suggesting that AVP neurons are exposed to ER stress even under basal conditions. This is likely due to the need for AVP neurons to continuously synthesize large amounts of peptides in the ER for the maintenance of water homeostasis. Taken together, this suggests that AVP neurons are vulnerable to ER stress, and that BiP is pivotal in maintaining the AVP neuron system.

ER stress, UPR and apoptosis are known to be closely related^33^. In almost all previous in vivo BiP whole-body and conditional knockout studies, apoptosis was reported to be involved in the cell death^34,35,37–42^, mainly based on TUNEL assay results. In contrast, we observed almost no TUNEL-positive cells in the SON and PVN of AVP neuron-specific BiP knockdown mice. Cell death has historically been classified into three different forms based on its morphological features: (1) type I cell death or apoptosis, manifesting as cytoplasmic shrinkage, chromatin condensation, nuclear fragmentation and plasma membrane blebbing; (2) type II cell death or autophagy, displaying extensive cytoplasmic vacuolization; and (3) type III cell death or necrosis, exhibiting no specific features of type I or II cell death^43^. Our observations of the ultrastructural morphology of dying AVP neurons revealed the emergence of autophagosome membranes and autolysosomes. In some cells, large vacuoles containing various organelles undergoing degradation were present throughout the cytoplasm. Furthermore, we did not find any morphological features characteristic of apoptosis or necrosis. These data suggest that autophagy was activated in AVP neurons during the cell death process induced by BiP knockdown. Investigation into the presence and absence of autophagic flux inhibitors can reveal the dynamic changes in autophagic processes^44^. In the current study, we showed that autophagic vacuoles were significantly increased in AVP neurons of AVP neuron-specific BiP knockdown mice under the pharmacological inhibition of autophagy by chloroquine treatment. These results demonstrate that autophagic flux was activated in AVP neurons after BiP knockdown.

The finding that autophagy was activated in AVP neurons following BiP knockdown-induced ER stress is consistent with our previous study showing that ER stress induces autophagy in organotypic cultures of the mouse hypothalamus^28^. ER stress-induced autophagy should primarily be a protective and adaptive mechanism by which misfolded/unfolded proteins and damaged organelles are cleared^45^. However, if excessive autophagy occurs over an extended period due to sustained ER stress, a greater proportion of organelles will be degraded, leading to the perturbation of cellular homeostasis and eventual cell death. This is exemplified in FNDI model mice, for which we previously reported that sustained ER stress caused by the accumulation of mutant AVP precursors in the ER results in the autophagy-associated cell death of AVP neurons^28^. In the present study, BiP knockdown in AVP neurons induced autophagy and AVP neuronal loss; however, pharmacological inhibition of autophagy by chloroquine exacerbated AVP neuronal loss. This indicates a protective role of autophagy in AVP neurons under conditions of ER stress.

Glial cells are the first elements that react to insults of the central nervous system^46^. Activated microglia migrate to and surround damaged or dead cells, clearing cellular debris and activating astrocytes through the production and release of pro-inflammatory cytokines^47^. In the current study, microglia and astrocytes were activated as indicated by the reactive gliosis that occurred in the SON and PVN of AVP neuron-specific BiP knockdown mice. Upregulation of the pro-inflammatory cytokines TNF-α, IL-6 and IL-1β was also observed in the PVN after BiP knockdown.

Astrocytes are known to be important components in the regulation of AVP release^48^. Hyperosmotic challenges retract the astrocytic processes surrounding AVP neurons, which facilitates neuronal interactions through synapses and dendritic gap junctions, increasing AVP neuronal excitability and promoting synchronous AVP secretion^49^. In contrast, hypoosmotic challenges inhibit AVP neuronal activity by evoking the expansion of astrocytic processes, wedging them in between neuronal dendrites and lessening dendritic gap junctional connectivity^49^. In the present study, AVP neuron-specific BiP knockdown led not only to the death of AVP neurons but also to the activation of astrocytes. While the increase in urine volume could be mainly attributed to the substantial loss of AVP neurons, activated astrocytes might also contribute to decreased AVP secretion from the residual AVP neurons, as reactive astrogliosis has been reported to induce subsequent perturbation of synaptic homeostasis and the neuronal-glial network^50,51^.

In conclusion, our data demonstrate that BiP knockdown in AVP neurons leads to ER stress and activates autophagy in AVP neurons followed by AVP neuronal loss, suggesting that BiP is essential for the AVP neuron system. In addition, autophagic inhibition exacerbates AVP neuronal loss due to BiP knockdown in AVP neurons, indicating a protective role of autophagy in AVP neurons under conditions of ER stress.

## Methods

### Animals

C57BL/6J mice were purchased from Chubu Science Materials (Nagoya, Japan). Mice were maintained under controlled conditions (23.0 ± 0.5 °C, lights on 09:00 to 21:00); male mice were used in all experiments. All procedures were approved by the Animal Experimentation Committee of the Nagoya University Graduate School of Medicine and performed in accordance with institutional guidelines for animal care and use.

### Viral vectors

The following viral vectors were cloned and produced as reported previously: rAAVs (serotype 1/2) carrying a conserved 1.9 kb AVP promoter followed by Venus cDNA (rAAV-AVPp-Venus) similar to previous studies^32,52^, mouse BiP shRNA cassette (rAAV-AVPp-BiP shRNA) from the Hspa5 Mouse shRNA Plasmid (TR500881; OriGene, Rockville, MD, USA) or scrambled shRNA cassette (rAAV-AVPp-scrambled shRNA)^53^. The sequences of the BiP shRNA were as follows: BiP shRNA1, 5′-CGATGTGTCTCTTCTCACCATTGACAATG-3′, BiP shRNA2, 5′-ATGTAATTGGAATCTTCACCTCAGAGTGG-3′, BiP shRNA3, 5′-TTCTACCATAAGTGACACCAATAAATGTT-3′, BiP shRNA4, 5′-ACCTATTCCTGCGTCGGTGTGTTCAAGAA-3′. Genomic titers of the viruses were determined with the QuickTiter AAV Quantitation Kit (Cell Biolabs, Inc., San Diego, CA, USA) and RT-PCR using the ABI 7700 cycler (Applied Biosystems, Waltham, MA, USA). The rAAV titers were between 10^9^–10^10^ genomic copies/μl. The efficacy of rAAV-AVPp-BiP shRNAs was compared, and rAAV-AVPp-BiP shRNA3 was selected as the most efficient. Therefore, rAAV-AVPp-BiP shRNA3 was employed in the following experiments.

### Stereotaxic targeting of rAAVs into the mouse SON and PVN

Two-month-old mice were anesthetized with 1–2% isoflurane (Wako, Osaka, Japan) using an animal anesthetization device (MA-AT210D, Muromachi Kikai, Tokyo, Japan) and placed on a stereotaxic apparatus (Model 900LS; Kopf Instruments, Tujunga, CA, USA). rAAV injections were performed using glass pipettes prepared from 1–5 μl micropipettes (708707; Brand, Wertheim, Germany) using a glass pipette puller (PC-100; Narishige, Tokyo, Japan). The injection volume of the rAAVs was 200 nl per nucleus. The injection coordinates were as follows, in accordance with the mouse brain atlas^54^: for the SON, A/P − 0.7 mm, M/L ± 1.25 mm, D/V − 5.4 mm; for the PVN, A/P − 0.8 mm, M/L ± 0.25 mm, D/V − 4.6 mm.

### Antibodies

The primary antibodies used for immunohistochemistry in the current study included: mouse anti-neurophysin II (AVP-NP) [1:100; PS41; kindly provided by Dr. Harold Gainer, National Institutes of Health (NIH), Bethesda, MD, USA]^55,56^, guinea pig anti-AVP (1:2000; T-5048; Peninsula, San Diego, CA, USA), mouse anti-neurophysin I (OT-NP; 1:100; PS38; a gift from Dr. Harold Gainer)^55,56^, chicken anti-GFP (1:10,000; ab13970; Abcam, San Diego, CA, USA), rabbit anti-IBA1 (1:200; ab178846; Abcam) and rabbit anti-GFAP (1:200; ab7260; Abcam). The following secondary antibodies were used in the present study: for immunofluorescence staining, Alexa Fluor 488-conjugated goat anti-chicken IgY (H + L) (1:1000; A11039; Invitrogen, San Diego, CA, USA), Alexa Fluor 546-conjugated donkey anti-mouse IgG (H + L) highly cross-adsorbed (1:1000; A10036; Invitrogen), Cy3-conjugated affinipure donkey anti-guinea pig IgG (H + L) (1:500; 706-165-148; Jackson ImmunoResearch, West Grove, PA, USA), Alexa Fluor 647-conjugated donkey anti-mouse IgG (H + L) highly cross-adsorbed (1:1000; A31571; Invitrogen) and Alexa Fluor 647-conjugated donkey anti-rabbit IgG (H + L) highly cross-adsorbed (1:1000; A31573; Invitrogen); for immunoelectron microscopy, biotinylated horse anti-mouse IgG (H + L) (1:200; BA-2000; Vector Laboratories, Burlingame, CA, USA).

### Immunohistochemistry

As described previously^28–30^, mice were deeply anesthetized and transcardially perfused with a cold fixative containing 4% paraformaldehyde (PFA) in phosphate-buffered saline (PBS), pH 7.4. After fixation, brains were removed and immersed in the same fixative for 24 h at 4 °C, then dissected and cut into 50-μm sections on a vibratome (VT1200 S; Leica Biosystems, Wetzlar, Germany). The sections were washed with PBS and 0.3% Triton X-100 in PBS, followed by blocking with a mixture of 5% normal goat serum and 3% bovine serum albumin in PBS for 1 h at room temperature (RT). For immunofluorescence staining, the sections were incubated with primary antibodies overnight at 4 ℃. After being rinsed in PBS with 0.05% Tween 20, slices were treated with corresponding secondary antibodies for 2 h at RT. Fluorescence images were acquired with a laser-scanning confocal microscope (LSM 5 Pascal; Carl Zeiss, Oberkochen, Germany) or a fluorescence microscope (BZ-9000; Keyence, Osaka, Japan) and processed using Adobe Photoshop CS5 (Adobe Systems, San Jose, CA, USA). For the TUNEL assay, the Apoptosis in situ Detection Kit (Wako) was used according to the manufacturer’s instructions.

### In situ hybridization and quantification

The BiP exonic probe was produced from a 922-bp fragment containing bases 852 to 1773 of the mouse BiP cDNA as described previously^31^. A radiolabeled antisense probe for BiP mRNA was synthesized from 1 μg of linearized template using 55 μCi [^35^S]UTP and 171 μCi [^35^S]CTP (PerkinElmer Life Sciences, Waltham, MA, USA) and the Riboprobe Combination System (Promega, Madison, WI, USA). After incubation for 1 h at 42 °C, the cDNA template was digested with DNase for 10 min at 37 °C. The radiolabeled RNA probe was purified using Quick Spin Columns for radiolabeled RNA purification (Roche Diagnostics, Basel, Switzerland), precipitated with ethanol, and resuspended in 100 μl of 10 mM Tris-HCl, pH 7.5 containing 20 mM dithiothreitol.

Mice were deeply anesthetized and transcardially perfused with cold 4% PFA in PBS, then brains were removed and immersed in the same fixative for 3 h at 4 °C, followed by cryoprotection in PBS containing 10–20% sucrose at 4 °C. Cryoprotected brains were then embedded in Tissue-Tek O.C.T. compound (Sakura Finetechnical, Tokyo, Japan) and stored at − 80 °C until sectioning. For sectioning, brains were cut into 16-μm sections on a cryostat (CM3050 S; Leica Biosystems) at − 20 °C, thaw-mounted on Superfrost Plus microscope slides (Matsunami Glass Ind., Osaka, Japan), and slices were stored at − 80 °C until in situ hybridization. Prehybridization, hybridization, and post-hybridization procedures were performed as described previously^26^. The sections were exposed to BioMax MR film (Carestream Health, Rochester, NY, USA) for 4 days. Expression levels in the SON and PVN were quantified by measuring the integrated optimal densities (optical densities × area) from the film images using ImageJ software (NIH).

### Measurements of urine volume, AVP concentration and plasma osmolality

Mice were housed in metabolic cages, and 24-h pooled urine was collected and measured throughout the experimental period. Urine AVP concentrations were measured with a radioimmunoassay kit (AVP kit YAMASA; Yamasa, Chiba, Japan). Blood was collected from the submandibular vein and immediately centrifuged for plasma separation. Plasma osmolality was determined using the cryoscopic method (Oriental Yeast Co., Ltd, Tokyo, Japan).

### Quantification of AVP neurons

The best-matched slices for the SON and PVN at 0.70 and 0.82 mm caudal from the bregma, respectively, in accordance with the mouse brain atlas^54^, were selected from each mouse for the analyses. The number of cells labeled with an anti-AVP-NP antibody by immunohistochemistry were counted per SON and PVN, and the mean values for each mouse were subjected to statistical analyses.

### Electron microscopy

As described previously^28–30^, mice were deeply anesthetized and transcardially perfused with 4% PFA and 2% glutaraldehyde (GA) in PBS. Brains were then immersed in the same fixative for 3 h at 4 °C. After fixation, brains were cut into 100-μm sections on a vibratome (VT1200 S). Free-floating sections were washed with 0.1 M phosphate buffer (PB) and 0.1% Triton X-100 in PB, followed by incubation with a mouse anti-AVP-NP antibody (PS41) overnight at 4 °C. Sections were then washed with 0.1 M PB and incubated with biotinylated horse anti-mouse IgG (H + L) (1:200) for 2 h at RT. Sections were washed then treated with avidin-biotin complex solution (1:100; Vectastain ABC-HRP kit; PK-4000; Vector Laboratories) for 90 min at RT. Signals were developed with 0.1 M PB containing 0.1% 3,3’-diaminobenzidine dihydrochloride (Sigma-Aldrich, St. Louis, MO, USA) and 0.004% hydrogen peroxide. The stained sections were further fixed in 2.5% GA in 0.1 M PB overnight at 4 °C, followed by post-fixation with 2% osmium tetroxide for 20 min at 4 °C. Each section was dehydrated in a graded ethanol series, treated with propylene oxide and embedded in epoxy resin (TAAB 812 resin; TAAB Laboratories Equipment, Aldermaston, UK). The resin was polymerized for 48 h at 60 °C. Ultrathin sections (70-nm thickness) including the SON were prepared using an ultramicrotome with a diamond knife (Reichert Ultracut S; Leica Biosystems) and counterstained with lead citrate before analysis with an electron microscope (JEM-1400EX; JEOL, Tokyo, Japan). For quantification of the ER, the total ER area was analyzed using immunoelectron microscopic images from 5 AVP neurons in each group. The area of the ER relative to that of the cytoplasm was determined from digitized images using ImageJ software. Autophagic vacuoles were counted using immunoelectron microscopic images^44^ from 7 to 8 AVP neurons in each group, and results were expressed per 10 µm^2^ of the cytoplasm.

### Quantitative real-time RT-PCR

Mice were sacrificed by cervical dislocation, and brains were immediately dissected followed by PVN isolation. The samples were frozen in liquid nitrogen and stored at − 80 °C until RNA extraction. Total RNA was extracted using TRIzol (Invitrogen) and the RNeasy kit (QIAGEN, Hilden, Germany). One microgram of total RNA was reverse-transcribed with ReverTra Ace qPCR RT Kit (Toyobo, Osaka, Japan). Quantitative real-time PCR reactions were performed using Power SYBR Green PCR Master Mix (Applied Biosystems) to assess relative mRNA levels of CHOP, spliced XBP1, TNF-α, IL-6 and IL-1β. As an internal standard control, 18S rRNA expression was simultaneously quantified. The following primer sequences were used in the current study: CHOP mRNA, 5′-CACCACACCTGAAAGCAGAA-3′ (forward), 5′-CGTTTCCTGGGGATGAGATA-3′ (reverse), spliced XBP1 mRNA, 5′-CTGAGTCCGAATCAGGTGCAG-3′ (forward), 5′-GTCCATGGGAAGATGTTCTGG-3′ (reverse), TNF-α mRNA, 5′-CATCTTCTCAAAACTCGAGTGACAA-3′ (forward), 5′-TGGGAGTAGATAAGGTACAGCCC-3′ (reverse), IL-6 mRNA, 5′-GTGGCTAAGGACCAAGACCA-3′ (forward), 5′-GGTTTGCCGAGTAGACCTCA-3′ (reverse), IL-1β mRNA 5′-TACAAGGAGAGACAAGCAACGACA-3′ (forward), 5′-GATCCACACTCTCCAGCTGCA-3′ (reverse) and 18S rRNA, 5′-TTGACGGAAGGGCACCACCAG-3′ (forward), 5′-GCACCACCACCCACGGAATCG-3′ (reverse). Relative mRNA expression was calculated using the comparative Ct method, and analyses were performed using the CFX Maestro qPCR system (Bio-Rad, La Jolla, CA, USA).

### Chloroquine administration

Two-month-old mice were treated with an intraperitoneal administration of chloroquine (20 mg/kg/day, Sigma-Aldrich) daily for 2 or 4 weeks, beginning just after the injection of rAAV-AVPp-scrambled shRNA or rAAV-AVPp-BiP shRNA. Control mice received a similar volume of vehicle without chloroquine. The dosage of chloroquine employed in this study was determined based on previous studies^57,58^.

### Statistical analysis

The statistical significance of the differences among groups was analyzed either by an unpaired Student’s *t*-test, one-way ANOVA or two-way ANOVA, with repeated measures followed by a Bonferroni test as appropriate. Results are expressed as the means ± SE, and differences were considered statistically significant at a value of *P* < 0.05.

## Supplementary information

Supplementary information 1.

Supplementary information 2.
