# Supplementary material for: Endoplasmic reticulum chaperone BiP/GRP78 knockdown leads to autophagy and cell death of arginine vasopressin neurons in mice
Source: Sci Rep. 2020 Nov 12;10:19730. doi: 10.1038/s41598-020-76839-z (PMC7661499; doi:10.1038/s41598-020-76839-z)

Endoplasmic reticulum chaperone BiP/GRP78 knockdown leads to autophagy and cell death of arginine vasopressin neurons in mice

Yohei Kawaguchi[1], Daisuke Hagiwara[1,2,*], Takashi Miyata[1], Yuichi Hodai[1], Junki Kurimoto[1], Hiroshi Takagi[1], Hidetaka Suga[1], Tomoko Kobayashi[1], Mariko Sugiyama[1], Takeshi Onoue[1], Yoshihiro Ito[1], Shintaro Iwama[1], Ryoichi Banno[1,3], Valery Grinevich[2] & Hiroshi Arima[1,*]

[1]Department of Endocrinology and Diabetes, Nagoya University Graduate School of Medicine, Nagoya, 466-8550, Japan

[2]Department of Neuropeptide Research in Psychiatry, Central Institute of Mental Health, Medical Faculty Mannheim, University of Heidelberg, 68159 Mannheim, Germany

[3]Research Center of Health, Physical Fitness and Sports, Nagoya University, Nagoya 464-8601, Japan

*Corresponding authors

Daisuke Hagiwara, MD, PhD

Department of Endocrinology and Diabetes, Nagoya University Graduate School of Medicine

65 Tsurumai-cho, Showa-ku, Nagoya, 466-8550, Japan

E-mail address: d-hagiwara@med.nagoya-u.ac.jp

Hiroshi Arima, MD, PhD

Department of Endocrinology and Diabetes, Nagoya University Graduate School of Medicine

65 Tsurumai-cho, Showa-ku, Nagoya, 466-8550, Japan

E-mail address: arima105@med.nagoya-u.ac.jp

Supplementary Figure 1.

Evaluation of apoptosis in AVP neuron-specific BiP knockdown mice.

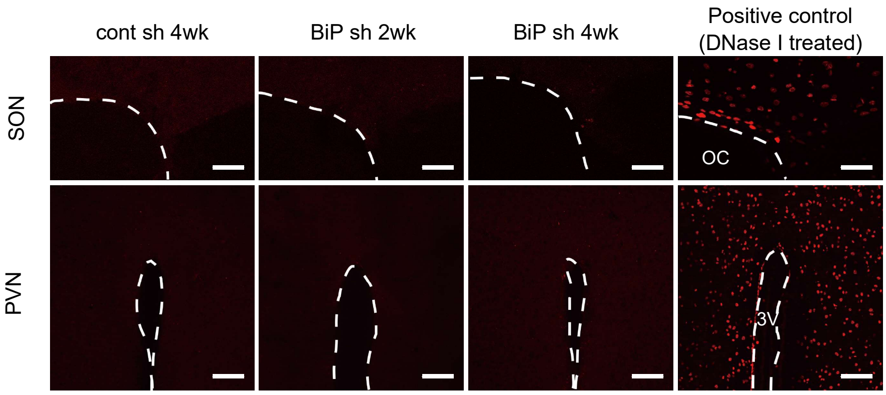

Supplementary Figure 2.

Evaluation of gliosis in AVP neuron-specific BiP knockdown mice.

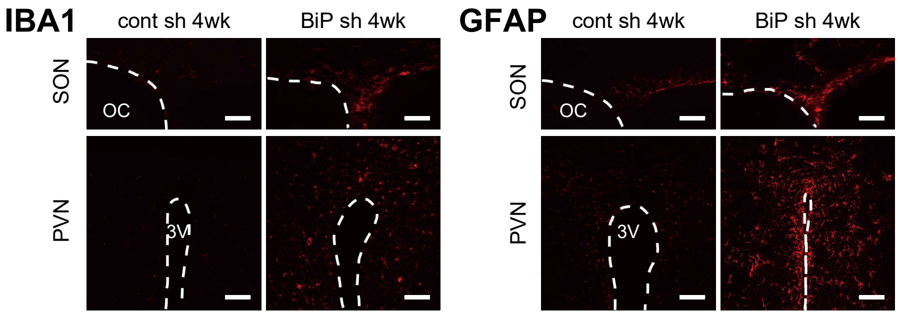

Supplementary Figure 3.

Effects of AVP neuron-specific BiP knockdown on the expression levels of pro-inflammatory cytokines.

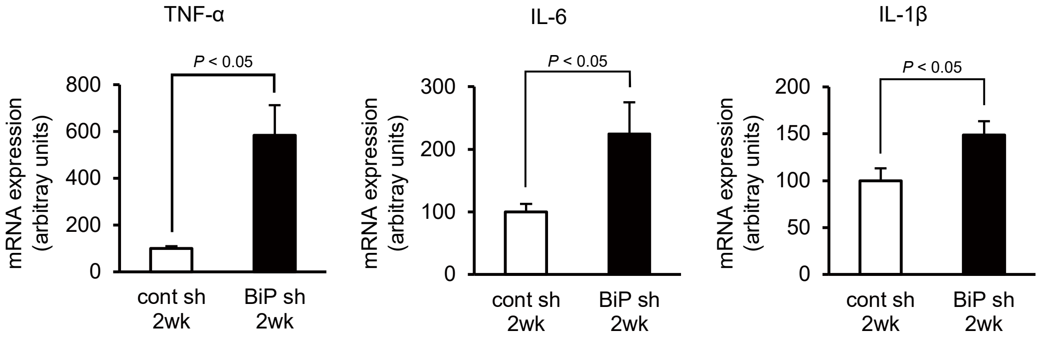

Supplement: Supplementary file 1 — Supplementary information 1. [file 41598_2020_76839_MOESM1_ESM.pdf]
