# Supplementary material for: Endoplasmic reticulum chaperone BiP/GRP78 knockdown leads to autophagy and cell death of arginine vasopressin neurons in mice
Source: Sci Rep. 2020 Nov 12;10:19730. doi: 10.1038/s41598-020-76839-z (PMC7661499; doi:10.1038/s41598-020-76839-z)
Supplement: Supplementary file 2 — Supplementary information 2. [file 41598_2020_76839_MOESM2_ESM.docx]

**Supplementary Figure 1.** Evaluation of apoptosis in AVP neuron-specific BiP knockdown mice. Representative TUNEL assay images from the SON and PVN four weeks after injection of rAAV-AVPp-scrambled shRNA (cont sh 4wk) or two (BiP sh 2wk) and four (BiP sh 4wk) weeks after rAAV-AVPp-BiP shRNA injection; some slices were treated with DNase I according to the manufacturer’s instructions as positive controls (positive control). OC, optic chiasm; 3V, third ventricle. Scale bars: 50 μm (SON), 100 μm (PVN).

**Supplementary Figure 2.** Evaluation of gliosis in AVP neuron-specific BiP knockdown mice. Fluorescence staining for IBA1 and GFAP in the SON and PVN four weeks after injection of rAAV-AVPp-scrambled shRNA (cont sh 4wk) or rAAV-AVPp-BiP shRNA (BiP sh 4wk). OC, optic chiasm; 3V, third ventricle. Scale bars: 50 μm (SON), 100 μm (PVN).

**Supplementary Figure 3.** Effects of AVP neuron-specific BiP knockdown on the expression levels of pro-inflammatory cytokines. Quantitative real-time RT-PCR analysis for TNF-α, IL-6 and IL-1β mRNA in the PVN two weeks after injection of rAAV-AVPp-scrambled shRNA (cont sh 2wk) and rAAV-AVPp-BiP shRNA (BiP sh 2wk). Mean mRNA expression levels in the cont sh 2wk group are expressed as 100. Results were analyzed by an unpaired Student’s *t*-test and are expressed as the means ± SE (*n* = 7 per group).
